# Level of inflammatory cytokines in rheumatoid arthritis patients: Correlation with 25-hydroxy vitamin D and reactive oxygen species

**DOI:** 10.1371/journal.pone.0178879

**Published:** 2017-06-08

**Authors:** Somaiya Mateen, Shagufta Moin, Sumayya Shahzad, Abdul Qayyum Khan

**Affiliations:** 1 Department of Biochemistry, Faculty of Medicine, Jawaharlal Nehru Medical College, Aligarh Muslim University, Aligarh, Uttar Pradesh, India; 2 Department of Orthopaedic Surgery, Faculty of Medicine, Jawaharlal Nehru Medical College, Aligarh Muslim University, Aligarh, Uttar Pradesh, India; Mayo Clinic Rochester, UNITED STATES

## Abstract

**Background:**

Rheumatoid arthritis (RA) is an autoimmune inflammatory disorder. Reactive oxygen species (ROS) and pro-inflammatory cytokines have been believed to be involved in the etiopathogenesis of the disease. The aim of the study was to determine the correlation of inflammatory cytokines with 25-hydroxy vitamin D and ROS.

**Methods:**

100 RA patients and 50 healthy age and sex matched individuals were included in the study. Patients were further divided on the basis of presence or absence of rheumatoid factor and disease severity. Serum 25-hydroxy vitamin D levels were monitored by chemiluminescent immunoassay. 10% hematocrit was used to detect the level of ROS by spectro fluorometer. The levels of inflammatory cytokines (TNF-α, IL-1β, IL-6, IL-10 and IL-17) were determined in plasma by ELISA.

**Results:**

The level of 25-hydroxy vitamin D was found to be decreased in RA patients in comparison to the control group. However the level of ROS and inflammatory cytokines were found to be elevated in RA patients in comparison with the healthy controls, with the increase being more pronounced in seropositive and RA patients having high disease severity. Inflammatory cytokines showed negative correlation with 25-hydroxy vitamin D and positive correlation with ROS.

**Conclusion:**

This study for the first time shows the association of inflammatory cytokines with 25-hydroxy vitamin D and ROS in RA patients. The results suggest that 25-hydroxy vitamin D being an immune modulator is decreased in the serum of RA patients. Further ROS and cytokines play an important role in the pathogenesis of RA and are responsible for increasing the severity of disease.

## Introduction

Rheumatoid arthritis (RA) is a chronic, inflammatory and autoimmune disease having a prevalence of approximately 0.5–1% of the population of industrialized world [1]. It is characterized by persistent synovial inflammation leading to joint damage and subsequent disability. Women are 3 times more prone to RA than men. Production of autoantibodies such as rheumatoid factor (RF) and anti-citrullinated peptide antibody, is one of the laboratory feature of RA [2].

The etiopathogenesis of RA remains incompletely understood, with the involvement of more than one mechanism in the disease development. 25-hydroxy vitamin D is not only involved in the bone and calcium metabolism but it is also considered as one of the important environmental factor relevant to many autoimmune diseases, including RA [3]. It has been reported to have immune-modulatory effects and greater intake of 25-hydroxy vitamin D has been linked with lower risk of RA. Vitamin D is also responsible for the regulation of redox signaling pathway thus controlling the reactive oxygen species (ROS) formation [4]. In addition, it also regulates inflammation by influencing both innate and adaptive arms of immunity [5].

Synovial fibroblasts and activated immune cells are responsible for the production of large number of inflammatory cytokines which are believed to play a crucial role in development and progression of RA [6]. Earlier RA was believed to be Th1 cell mediated autoimmune disease but now Th17 cells are also considered to be fundamental in the disease process [7, 8].

The characteristic inflammation of RA occurs due to the abundance of inflammation promoting cytokines over those of the inhibiting ones [9]. Among the pro-inflammatory cytokines, TNF-α is the principal cytokine which regulates the formation of other inflammatory mediators in the synovial tissue [10]. It is also involved in the destruction of bone and cartilage via the activation of chondrocytes and osteoclasts [11]. IL-1 and IL-6 are the other key cytokines involved in the pathogenesis of RA. IL-17A and IL-17F are the signature cytokines of Th17 cells [12]. These pro-inflammatory cytokines are also responsible for the formation of chemokines, matrix-metalloproteases (MMPs), inducible nitric oxide synthase, osteoclasts differentiation and the expression of cell adhesion molecules [9]. There occurs a compensatory response in the RA synovium due to presence of anti-inflammatory cytokines such as IL-4, IL-5 and IL-10 [13].

ROS have also been implicated in the pathogenesis of RA. These reactive species acts as second messenger in the activation of nuclear factor kappa-B (NF-κB) thereby allowing transcription of various inflammatory mediators [14]. The overproduction of ROS is responsible for the oxidation of biomolecules. Literature suggests the weakening of antioxidant defence system and subsequent over-production of oxidants thereby leading to oxidative stress in RA patients [15–17]. So there occurs a complex interplay between ROS and inflammatory mediators.

The aim of the present study was to evaluate the level of 25-hydroxy vitamin D, ROS and inflammatory cytokines in the blood of RA patients and to find the association of inflammatory mediators with 25-hydroxy vitamin D and ROS. These parameters were also compared in patients further divided on the basis of disease severity and the presence or absence of RF. To the best of our knowledge this study for the first time shows the correlation of inflammatory cytokines with 25-hydroxy vitamin D and ROS.

## Methods

### Subjects

This study was conducted on 100 RA patients (24–60 years) who visited the orthopaedics OPD of Jawaharlal Nehru Medical College, Aligarh. All the patients included in the study were newly diagnosed and they fulfilled the European League Against Rheumatism (EULAR) 2010 classification criteria for RA [18]. Control group consisted of 50 healthy age (23–60 years) and sex matched individuals. The study was carried out between June 2015 and December 2016. Written informed consent was obtained from all the blood donors prior to the withdrawal of blood. The study was approved by the Institutional Ethics and Research Advisory Committee, Faculty of Medicine, Jawaharlal Nehru Medical College, Aligarh Muslim University, India. Patients included in the study were not suffering from any chronic disease nor were alcoholics nor smokers. All the blood donors were not taking any additional vitamin supplement. The characteristics of controls and RA patients is given in Table 1.

**Table 1:** Baseline characteristics of healthy controls and rheumatoid arthritis patients. The values represents the mean ± S.D.

	Controls	RA patients
**Age**	40.06±10.63	40.92±10.24
**Height (cm)**	161.9±4.31	163.03±4.72
**Weight (Kg)**	58.14±5.18	59.4±4.61
**BMI**	22.1±1.46	22.34±1.35
**ESR (mm/hr)**	12.96±5.75	28.03±6.49*

*p<0.05

### Disease severity measurement

Disease severity was assessed by calculating 28-Joint Count disease activity score (DAS28). It uses number of swollen and tender joints, erythrocyte sedimentation rate (ESR) and patient’s health assessment by using visual analogue scale (VAS) [19]. DAS 28 ≤3.2 corresponds to low disease severity, DAS28>3.2≤5.1 corresponds to moderate disease severity while DAS28>5.1 represents high disease severity.

### Blood samples

Blood from the RA patients and healthy controls was taken in anti-coagulant vial. Plasma was used for the estimation of cytokines. After storing the plasma buffy coat was removed and the erythrocytes were washed thrice with phosphate buffered saline (pH 7.2). 10% haematocrit was used for quantifying the ROS level. Hemolysate was prepared as described previously [15].

### 25-hydroxy vitamin D Assay

The serum level of 25-hydroxy vitamin D was evaluated by automated analyser based on chemiluminescent immunoassay (Beckman Coulter—Access 2).

### Determination of ROS

2’, 7’- Dichlorofluorescein diacetate (DCF-DA) was used to measure intracellular ROS production by the method described by Keller et al [20]. Excitation and emission wavelength of 485 and 530 nm respectively was used to record fluorescence intensity by using spectrofluorometer.

### Determination of NO level

The amount of NO in the plasma was estimated by the method described by Miranda et al [21].

### Determination of reduced glutathione (GSH)

Method described by Jollow was used to estimate glutathione level in the hemolysate [22].

### Cytokine assays

The plasma was used to detect the levels of TNF-α and IL-1β (R&D Systems, USA) by following the manufacturer’s protocol. Human ELISA kits from Diaclone, France were used to detect the levels of IL-6, IL-10 and IL-17A by following the manufacturer’s protocol. The sensitivity of the ELISA kits for the detection of TNF-α, IL-1β, IL-6, IL-10, IL-17A was 5.5pg/ml, 1pg/ml, < 2pg/ml, < 5pg/ml, < 2.3pg/ml respectively.

### Statistical analysis

Data is represented as mean ±S.D. Data distribution was checked with Shapiro–Wilk test. Depending upon the result student’s t-test or Man-Whitey test was used to assess the difference between the two groups. Correlation analysis of cytokines with 25-hydroxy vitamin D and ROS was done by using Spearman’s rank correlation test. P values of less than 0.05 were considered statistically significant.

## Results

There was no statistical difference in age and BMI between healthy controls and RA patients.

**Fig 1:**
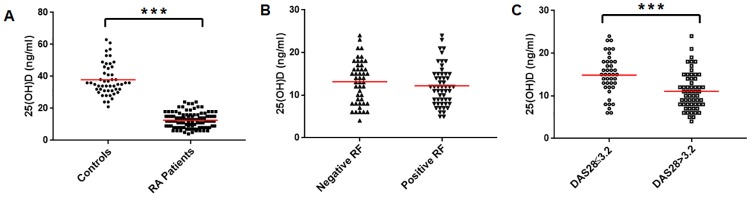
Serum level of 25-hydroxy vitamin D in controls and RA patients (Fig A), seropositive and seronegative RA patients (Fig B) and patients having DAS28≤3.2 and those having DAS28>3.2 (Fig C). (***p<0.001).

The level of 25-hydroxy vitamin D was found to be significantly reduced in RA patients (12.66±4.81 ng/ml) in comparison to the healthy controls (37.88±9.78 ng/ml) (Fig 1A). Patients having DAS28 >3.2 had lower 25-hydroxy vitamin D level (11.07±4.31 ng/ml) in comparison to patients having low disease activity (14.86± 4.63 ng/ml) (Fig 1B). However there was no statistical difference in the levels of 25-hydroxy vitamin D in seropositive (12.21±4.62 ng/ml) and seronegative RA patients (13.17± 5.01 ng/ml) (Fig 1C).

**Fig 2:**
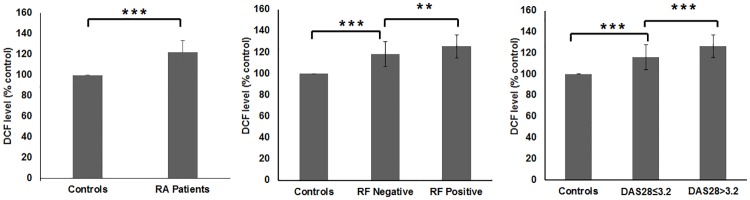
Bar graph representation of data from DCFH-DA assay on 10% hematocrit of control and RA patients (Fig A), seropositive and seronegative RA patients (Fig B) and patients having DAS28≤3.2 and those having DAS28>3.2 (Fig C). (**p<0.01, ***p<0.001).

The level of ROS in RA patients was found to be significantly increased in comparison to the healthy control (Fig 2A). Patients having DAS28 >3.2 had higher ROS level relative to the patients having DAS28≤ 3.2 (Fig 2B). Moreover seropositive RA patients also displayed higher ROS formation in comparison to the seronegative RA patients (Fig 2C).

The levels of cytokines and biochemical markers in patients sub-grouped according to the presence or absence of rheumatoid factor and DAS are shown in Table A and Table B in S1 file.

**Fig 3:**
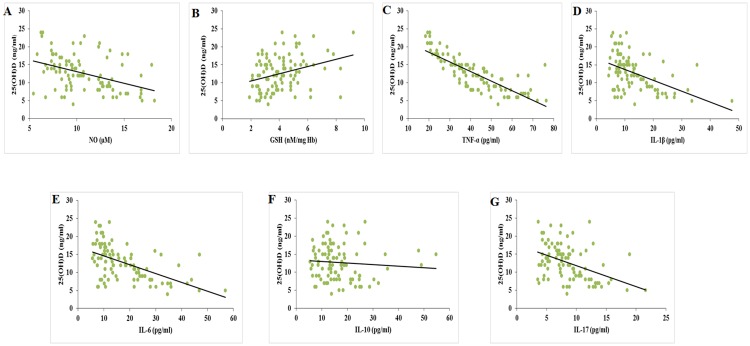
Correlation between 25 (OH) D and NO (Fig A), GSH (Fig B), TNF-α (Fig C), IL-1β (Fig D), IL-6 (Fig E), IL-10 (Fig F) and IL-17 (Fig G).

**Fig 4:**
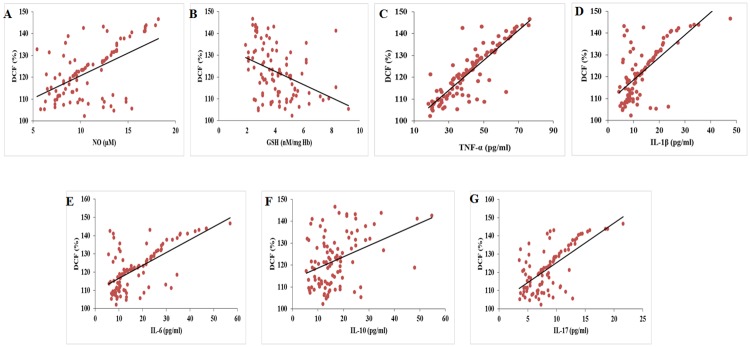
Correlation between ROS and NO (Fig A), GSH (Fig B), TNF-α (Fig C), IL-1β (Fig D), IL-6 (Fig E), IL-10 (Fig F) and IL-17 (Fig G).

Correlation analysis revealed that there occurs a negative association between 25-hydroxy vitamin D—cytokines and NO (Fig 3). However GSH showed positive correlation with vitamin D. ROS showed positive association with the inflammatory cytokines and NO while GSH was found to be negatively associated with ROS (Fig 4) (Table 2).

**Table 2:** Correlation analysis of plasma levels of cytokines with 25-hydroxy vitamin D and reactive oxygen species.

Cytokine	25(OH)D	ROS
TNF-α	-0.9*	0.8*
IL-1β	-0.5*	0.6*
IL-6	-0.6*	0.6*
IL-10	-0.2	0.4*
IL-17	-0.5*	0.6*
NO	-0.4*	0.5*
GSH	0.3*	-0.5*

*p<0.05

## Discussion

This is the first study showing the correlation of inflammatory cytokines with 25-hydroxy vitamin D and ROS levels in RA patients. The level of Th1, Th2 and Th17 cytokines were found to be higher in RA patients seropositive for RF and those having high disease severity in comparison to seronegative and low disease severity patients.

**Fig 5:**
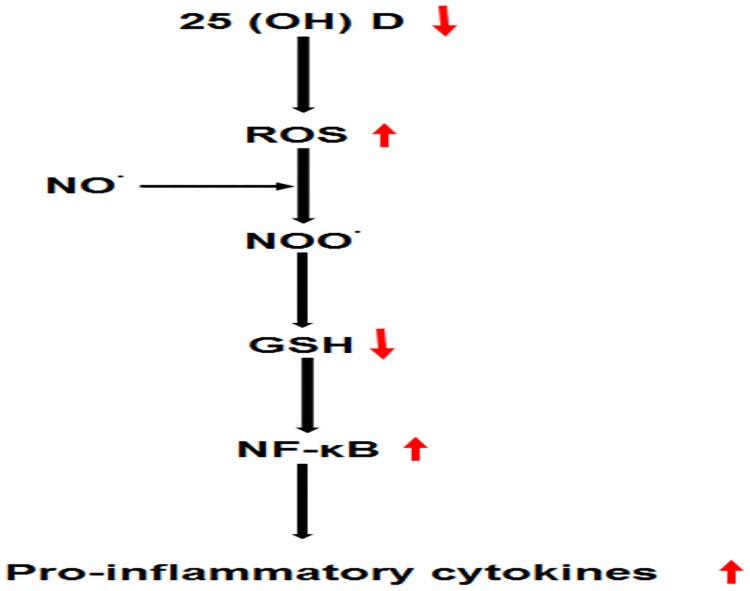
Schematic diagram showing 25 (OH) D mediated production of inflammatory cytokines. 25 (OH) D regulates the formation of reactive oxygen species, therefore a decrease in 25 (OH) D favours the ROS formation. ROS along with nitric oxide forms highly reactive peroxynitrite which has the potential to deplete antioxidants. As a result redox balance is disturbed thus favouring the formation of pro-inflammatory cytokines due to activation of signalling pathways (NF-κB).

25-hydroxy vitamin D acts as an immunemodulator by interacting with its intra-cytoplasmic receptor thereby significantly effecting immune cells [23]. Vitamin D is also responsible for maintaining low ROS level in the body [24]. Previous studies have also demonstrated a significant decrease in its level, with the decrease being more significant in patients having high disease activity [25]. It has been reported to inhibit the release of pro-inflammatory cytokines including TNF-α, IL-2 and interferon-γ [23]. The decreased level of 25-hydroxy vitamin D might be responsible for inducing inflammation in RA patients. The role of 25-hydroxy vitamin D in redox signalling also suggests that decreased 25-hydroxy vitamin D level increases the ROS formation. These reactive species reacts with nitric oxide thus forming highly reactive peroxynitrite, which in turn results in the depletion of antioxidants and oxidation of biomolecules. As a result redox balance is disturbed (decrease in reduced glutathione level) and signalling cascade (NF-κB) is activated which in turn further exacerbates the formation of cytokines in RA patients (Fig 5).

The over-production of ROS and pro-inflammatory cytokines are involved in the development and progression of many autoimmune diseases, including RA [15,26]. ROS has been reported to induce the oxidation of biomolecules (proteins, DNA and lipids) in synovial fluid and blood of RA patients [27, 28]. The lower levels of anti-oxidants have also been found in RA patients which in turn are responsible for increasing the ROS production [16, 29]. It also induces the depolymerisation of hyaluronic acid thus resulting in the decreased joint viscosity and increased bone resorption. The results of the present study shows that over-production of ROS is involved in increasing the severity of RA, as suggested by higher ROS level in patients having DAS > 3.2. Higher ROS level in seropositive RA patients reveals the role of rheumatoid factor in increasing the disease severity by inducing the formation of reactive intermediates.

Higher level of pro-inflammatory cytokines found in patients having DAS > 3.2 shows that inflammatory cytokines along with ROS, are involved in increasing the severity of RA by promoting oxidative stress and inflammation. Moreover the presence of rheumatoid factor further exacerbates the disease conditions in RA patients, as revealed by higher cytokines level in patients positive for RF than those found in seronegative RA patients.

In rheumatoid synovial tissue TNF-α is the principal cytokine which regulates the formation of other pro-inflammatory cytokines. Production of CTGF (connective tissue growth factor) is mediated by the activation of synovial fibroblasts by TNF-α. This in turn promotes the hyper activation of osteoclasts and thus the destruction of joints [30]. Previous studies have shown the higher TNF-α level in synovial fluid and serum of RA patients relative to the healthy controls [31]. TNF-α and IL-1β are the known activators of NF-κB cascade and they in turn creates a positive feedback loop [32]. TNF-α promotes the phosphorylation of inhibitor kinase beta thereby allowing the migration of NF-κB dimers to nucleus and thus allowing the transcription of inflammatory mediators. Activation of NADPH oxidase enzyme, which is responsible for the ROS generation, is also driven by TNF- α. It is also responsible for the formation of pannus (inflammatory vascular tissue formed over a joint surface of RA patients) by inducing the production of variety of chemokines and endothelial cell activation [14]. The production of pro-inflammatory cytokines has been found to be decreased by neutralizing the TNF- α in synovial cell cultures. Currently there are 5 anti-TNF- α agents (infliximab, etarnercept, adalimumab, certolizumab pegol, golimumab) approved by FDA for the treatment of RA patients [9].

IL-1β shares some pro-inflammatory activities with TNF-α such as the T cell activation and promotion of pro-inflammatory cytokines production [33]. However it is more crucial in destruction of cartilage than TNF-α and it does not promote the formation of TNF-α [34]. IL-1β has been found to be elevated in synovial fluid and plasma of RA patients [35]. It has been reported to be associated with disease activity such as the duration of morning stiffness [9]. Currently there is only one FDA approved anti-IL-1β agent, anakinra which is recombinant human antagonist of IL-1 receptor. Moreover Canakinumab, a monoclonal antibody against IL-1 has also entered clinical trials and it may prove useful for the RA treatment [10].

Elevated IL-6 level has been reported in the blood and synovial fluid of RA patients. It acts on neutrophils and releases proteolytic enzymes and reactive oxygen intermediates thus promoting inflammation and joint destruction [36]. This cytokine is associated with some laboratory findings such as the hypercoagulability, hypoalbuminemia and C-reactive protein (CRP) [37]. It has also been reported to be positively correlated with markers of inflammation in RA patients [38]. It is a more strong inducer of immunoglobulins and acute phase proteins production than TNF-α or IL-1. Serum levels of IL-6 has also been shown to be positively correlated with TNF-α [39]. Some anti-inflammatory effects of IL-6 has also been reported such as the inhibition of pro-inflammatory cytokine release and the stimulation of the production anti-inflammatory IL-1Ra [9]. Tocilizumab is the currently FDA approved anti-IL-6 drug while sarilumab and sirukumab are still in clinical trials [40].

Earlier RA was thought to be driven by Th 1 cells producing pro-inflammatory cytokines such as interferons, TNF and IL-2. However Th 17 cells have also been shown to be involved in the pathogenesis of RA [41]. These Th17 cells and IL-17 has been reported to be elevated in RA patients relative to the healthy controls [40]. In a study conducted by Kotake et al, IL-17 was not found to be correlated with DAS28, however higher IL-17 level was found in patients moderate DAS relative RA patients having low DAS [42]. Similar to the previous studies, our result also demonstrates elevated IL-17 level in plasma of RA patients, with the level being more elevated in high disease activity and seropositive RA patients. Th17 cells are also responsible for the production of other pro-inflammatory mediators such as IL-22, IL-26 and chemokine CCL20, apart from IL-17A and IL-17F. IL-17 along with IL-9 promotes the secretion of chemokine and cytokines which in turn accelerates the infiltration of neutrophils in the tissues thereby causing inflammation and injury [40]. The blockade of IL-17 has shown beneficial effects on murine arthritis so this blockade might be effective in the treatment of human RA. Certain drugs (such as ixekizumab, secukizumab, brodylumab) targeting IL-17A have entered clinical trials (stage III) [10].

IL-10 is an anti-inflammatory and immunoregulatory cytokine which supresses the formation of pro-inflammatory cytokines as well as it down regulates the functioning of antigen-presenting cells [16, 43]. It also inhibits the production of proteases and stimulates the formation of tissue inhibitor of metalloproteinases-1 (TIMP-1) by monocytes [44]. High levels of IL-10 detected in the plasma of RA patients is in agreement with previous studies [45]. A more significant increase in the level of IL-10 in patients having DAS > 3.2 shows that the level of anti-inflammatory IL-10 rises in response to higher inflammatory state of these patients. In addition, studies conducted on the animal model of arthritis reveals the beneficial effect of IL-10 on the reduction of severity of arthritis [44].

The main limitation of our study was that we could not find the correlation of vitamin D and ROS with many pro- and anti-inflammatory cytokines. Future studies depicting the correlation of other cytokines with vitamin D and ROS are needed to be done.

## Conclusion

This study for the first time demonstrates the correlation of plasma level of inflammatory cytokines with 25-hydroxy vitamin D and ROS in RA patients. Lower 25-hydroxy vitamin D and higher ROS level are involved in increasing the production of cytokines, with the increase being more pronounced in seropositive and patients having DAS > 3.2. These data further suggest that the ROS and inflammatory cytokines plays a crucial role in the pathogenesis of RA.

## Supporting information

S1 File Biochemical markers and inflammatory cytokines in RA patients. **Table A.** Comparison of biochemical markers and inflammatory cytokines in seronegative and seropositive RA patients. **Table B.** Comparison of biochemical markers and inflammatory cytokines in RA patients having DAS≤3.2 and those having DAS>3.2.
(PDF)
