# Supplementary material for: Level of inflammatory cytokines in rheumatoid arthritis patients: Correlation with 25-hydroxy vitamin D and reactive oxygen species
Source: PLoS One. 2017 Jun 8;12(6):e0178879. doi: 10.1371/journal.pone.0178879 (PMC5464597; doi:10.1371/journal.pone.0178879)
Supplement: S1 File — Table A. Comparison of biochemical markers and inflammatory cytokines in seronegative and seropositive RA patients. Table B. Comparison of biochemical markers and inflammatory cytokines in RA patients having DAS≤3.2 and those having DAS>3.2. (PDF) [file pone.0178879.s001.pdf]

Table A. Comparison of biochemical markers and inflammatory cytokines in seronegative and seropositive RA patients

Parameter	Seronegative	Seropositive
**NO (µM)**	9.89±2.89	11.4±3.07
**GSH (nmoles/mg Hb)**	4.79±1.7	3.55±0.99
**TNF-α (pg/ml)**	36.24±13.75	46.54±13.76
**IL-1β (pg/ml)**	10.56±6.38	16.05±7.67
**IL-6 (pg/ml)**	14.5±9.31	20.81±11.16
**IL-10 (pg/ml)**	15.43±8.14	18.17±9.45
**IL-17 (pg/ml)**	7.53±3.23	9.47±3.58

Table B. Comparison of biochemical markers and inflammatory cytokines in RA patients having DAS≤3.2 and those having DAS>3.2

Parameter	DAS≤3.2	DAS>3.2
**NO (µM)**	9.31±2.39	11.69±3.13
**GSH (nmoles/mg Hb)**	4.58±1.42	3.81±1.48
**TNF-α (pg/ml)**	35.1±12.25	46.48±14.43
**IL-1β (pg/ml)**	9.04±3.6	16.68±8.08
**IL-6 (pg/ml)**	11.52±5.55	22.42±11.32
**IL-10 (pg/ml)**	13.38±7.69	19.42±8.95
**IL-17 (pg/ml)**	6.92±2.19	9.75±3.86
